# Efficient All‐2D Amorphous Cobalt Sulfide Nanosheets/Multilayered Molybdenum Disulfide Hybrid Heterojunction Catalyst for Electrochemical Hydrogen Evolution

**DOI:** 10.1002/gch2.201900066

**Published:** 2019-12-09

**Authors:** Zhenbang Li, Pu Liu, Guowei Yang

**Affiliations:** ^1^ State Key Laboratory of Optoelectronic Materials and Technologies Nanotechnology Research Center School of Materials Science & Engineering Sun Yat‐sen University Guangzhou 510275 Guangdong P. R. China

**Keywords:** all‐2D catalysts, amorphous nanosheets, compound heterojunction structures, electrochemical hydrogen evolution, laser ablation

## Abstract

Hydrogen energy is considered to be a critical environmentally friendly and widely sourced renewable energy source that can be used as an alternative to fossil fuels. At present, the preparation of hydrogen (H_2_) mainly depends on traditional fossil fuels. In order to achieve sustainable development of environmental protection, great attention has been paid to the preparation of H_2_ by electrocatalysis, photocatalysis, and photoelectrochemistry. Here, it is reported for the first time that a novel active catalyst for the hydrogen evolution reaction, consisting of all‐2D amorphous nanosheets/2D crystal layer heterojunction structure and without any noble metal (no precious metals are present in the preparation or measuring), is almost entirely fabricated by laser ablation in liquid (LAL) growth of amorphous cobalt sulfide on the surface of multilayered molybdenum disulfide. In acidic media, the amorphous cobalt sulfide nanosheets/multilayered molybdenum disulfide (a‐CoS/MoS_2_) catalyst exhibits fast hydrogen evolution kinetics with onset potential of −147 mV and a Tafel slope of 126 mV per decade, which is much better than only the amorphous cobalt sulfide and molybdenum disulfide layer. The high hydrogen evolution activity of the amorphous cobalt sulfide nanosheets/multilayered molybdenum disulfide hybrid is likely due to the unique electrocatalytic synergistic effects between hydrogen evolution‐active amorphous cobalt sulfide nanosheets and layered crystal molybdenum disulfide materials, as well as the much‐increased catalytic sites. This work provides a new general route based on all‐2D amorphous nanosheets/2D crystal structure for designing and preparing novel layered materials with effectively manipulated catalytic properties and active functionality surface.

## Graphical abstract

A general strategy for fabricating all‐2D amorphous nanosheets/2D MoS_2_/CoSe_2_ hybrid heterojunction structure nanomaterials is described.

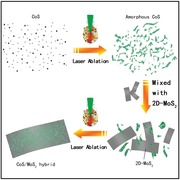

## 1 Introduction

With the increasing global energy demand, the fossil energy crisis and environmental pollution are becoming more serious. For this reason, researchers have paid more and more attention to the conversion methods and storage technologies of efficient and sustainable clean energy, such as light, electrolytic oxygen/hydrogen evolution by porous catalysts from water, fuel cells, and so on.^1^, ^2^, ^3^ Among them, hydrogen energy is generally considered to be an environmentally friendly and widely sourced alternative to fossil fuels. At present, the preparation of hydrogen (H_2_) mainly depends on traditional fossil fuels. In order to achieve sustainable development of environmental protection, great attention has been paid to the preparation of H_2_ by electrocatalysis, photocatalysis, or photoelectrochemical methods. Water is the ideal substance consumed by the reaction of oxygen evolution/hydrogen in photocatalytic or electrocatalytic processes. The produced H_2_ and O_2_ products can react again in the internal combustion engine or fuel cell to produce water, thus realizing the recycling of water. Therefore, the electrochemical hydrogen evolution reaction (HER) is very important as one of the semireactions of oxygen evolution/hydrogen evolution in the electrolytic water process. So, in the current world energy field, the development of high‐efficiency and low‐cost HER catalyst has become a more and more urgent need.

Recently, various MoS_2_ nanostructures, such as MoS_2_ nanoparticles, have been identified as a kind of the promising hydrogen evolution catalysts, while the shiny one among them is crystalline monolayered MoS_2_ structure.^4^ Furthermore, compared to MoS_2_ nanoparticles, the amorphous molybdenum sulfide films have been exhibited to have higher activity.^5^, ^6^, ^7^, ^8^ Thus, these understandings have led to many great efforts to develop interesting nanostructured MoS_2_‐based HER catalysts in order to maximize the number of edge sites, including amorphous materials^9^, ^10^ and MoS_2_‐based hybrid structures,^11^, ^12^, ^13^, ^14^, ^15^, ^16^ while the similar promoted performances have also been observed on Co‐based HER catalysts.^19^, ^20^, ^21^, ^22^, ^23^, ^24^ On the other hand, the synergetic chemical coupling effects between Co and Ni have been confirmed to have great contribution to the catalytic enhancement behavior,^19^ while the similar promoted performances have also been observed on Co–Mo–S*_x_* and carbon/metal‐sulfide catalytic materials.^12^, ^13^ Therefore, on the basis of these former researches, this work points to achieve a new and efficient HER catalysts based on an interesting preparation technology called laser ablation in liquid (LAL) growth process (which has been considered to be an efficient way to prepare amorphous nanosheets at room temperature^17^, ^18^) to fabricate a new construction of amorphous nanosheet‐2D crystal structure.

Here, we report for the first time that a novel HER electrocatalyst based on amorphous cobalt sulfide nanosheets/multilayered molybdenum disulfide hybrid heterojunction (denoted as a‐CoS/MoS_2_) is well prepared. It is highly active in acidic electrolyte, and notably, without any noble metals. The a‐CoS/MoS_2_ hybrid catalyst shows an onset potential much better than 2D‐MoS_2_ nanosheets and amorphous CoS, with a smaller Tafel slope of 126 mV per decade. These results suggest a new strategy for designing non‐noble metal catalysts with effectively manipulated and enhanced HER performance that can be comparable to the state‐of‐the‐art Pt‐based catalysts.

## 2 Results and Discussion

**Figure 1:**
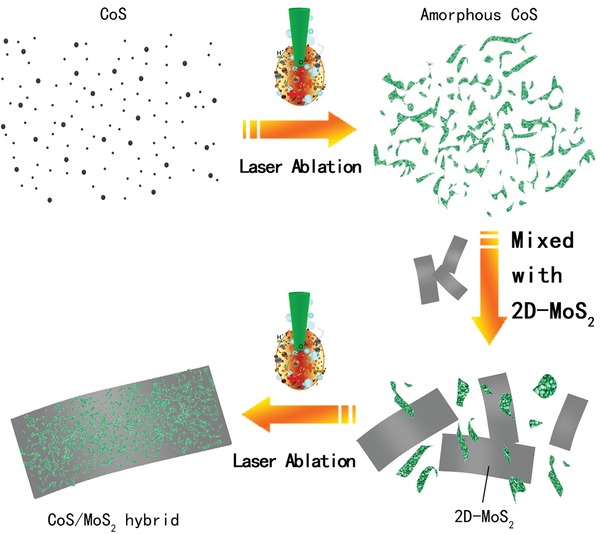
Schematic illustration of the preparation of MoS_2_/CoSe_2_ hybrid.

**Figure 2:**
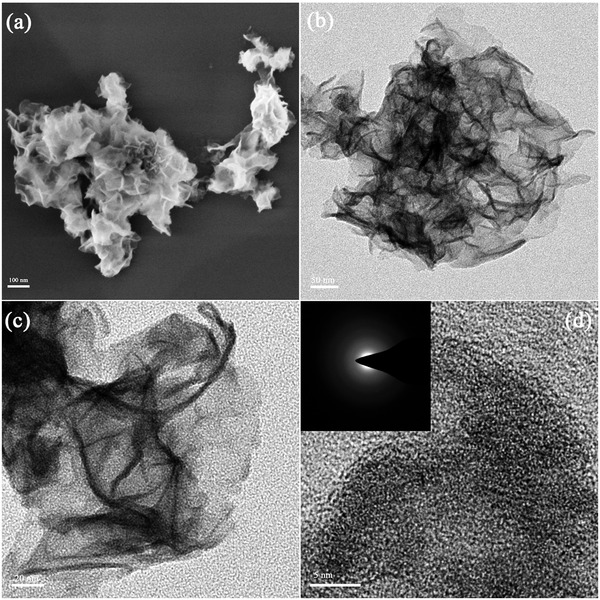
a) SEM image of Pre‐LAL CoS. b,c) TEM images of Pre‐LAL CoS. d) HRTEM image of pre‐LAL CoS. The inset shows the corresponding SAED pattern.

The amorphous CoS was first prepared by LAL, while the a‐CoS/MoS_2_ hybrid was also prepared directly by LAL (**Figure** **1**, see the Experimental Section for details of the synthesis). The 2D‐MoS_2_ (less than five layers) was first examined by scanning electron microscopy (SEM) and transmission electron microscopy (TEM, shown in Figure S1, Supporting Information), which revealed that the compact graphene‐like MoS_2_ has multilayered structure. The amorphous CoS and a‐CoS/MoS_2_ hybrid was definitely shown by means of SEM and TEM analysis (Figures 1 and **2**) respectively. For the structure and morphology detection of amorphous CoS nanosheets, a typical SEM image with low‐magnification in Figure 2a clearly indicated that the pre‐LAL samples were composed of numerous nanosheets with wrinkled surfaces. In addition, the TEM image (Figure 2b) and the representative high‐resolution TEM (HRTEM) analysis (Figure 2c,d) all further revealed that the CoS nanostructure has high‐density wrinkled surfaces consisting of thin nanosheets. Moreover, the corresponding selected‐area electron diffraction (SAED) pattern in the inset of Figure 2d shows a broad and diffused halo ring, which clearly suggested that the nanosheets have amorphous phase state.^17^

**Figure 3:**
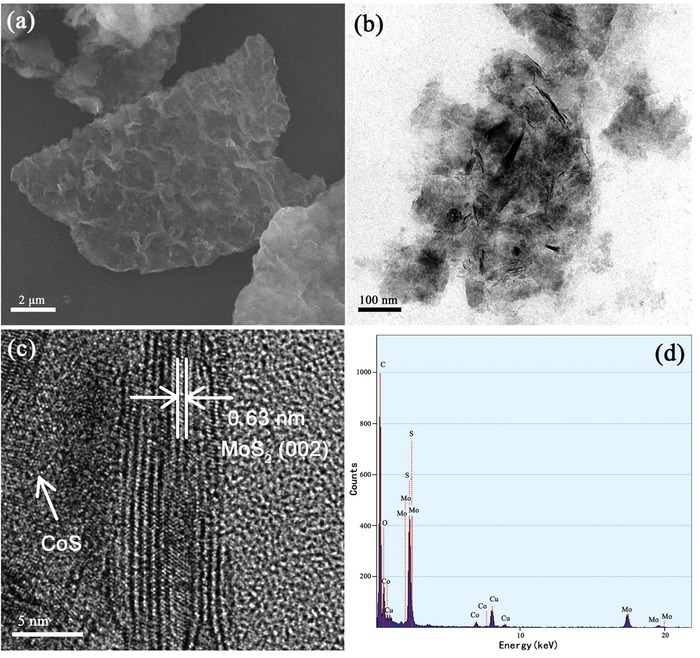
a) SEM image of CoS/MoS_2_ hybrid. b) TEM images of CoS/MoS_2_ hybrid. c) HRTEM image of CoS/MoS_2_ hybrid. d) EDS image of CoS/MoS_2_ hybrid.

**Figure 4:**
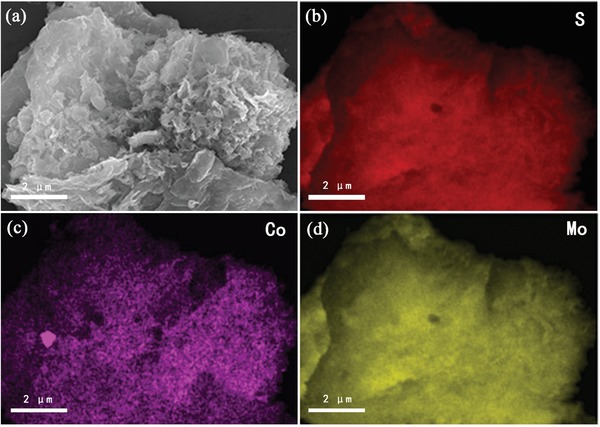
EDX elemental mapping of CoS/MoS_2_ hybrid showing clearly the homogeneous distribution. a) SEM image of CoS/MoS_2_ hybrid, b) S (red), c) Co (purple), and d) Mo (yellow).

**Figure** **3** shows the detailed structure and morphology detection of a‐CoS/MoS_2_ hybrid. Figure 3a presents an SEM image of the a‐CoS/MoS_2_ hybrid heterojunction structure. The obviously wrinkled surfaces of the a‐CoS/MoS_2_ hybrid suggested that MoS_2_ nanosheets were coated by amorphous CoS nanosheets. Figure 3b presents a TEM image of the CoS/MoS_2_ hybrid. As a comparison, the completely different morphology can be found on the MoS_2_ multilayered nanosheets (Figure S1, Supporting Information). Furthermore, the HRTEM image of a‐CoS/MoS_2_ hybrid (Figure 3c) revealed more details of the morphology prepared hybrid. The layered MoS_2_ nanosheets, with an interlayer separation of 0.63 nm, were observed in the hybrid, while the amorphous CoS nanosheets can be frequently found on the surface of MoS_2_ layer. In chemical composition analysis, energy‐dispersive X‐ray spectrum (EDX; Figure 3d) shows that both Co and Mo can be found on the hybrid nanosheets. In order to further confirm the formation of a‐CoS/MoS_2_ hybrid with Co, Mo, and S as its principal elemental components, EDX elemental mappings were measured and shown in **Figure** **4**, which revealed a uniform distribution of the Co, Mo and S over the whole detection range of the constructed hybrid material.

**Figure 5:**
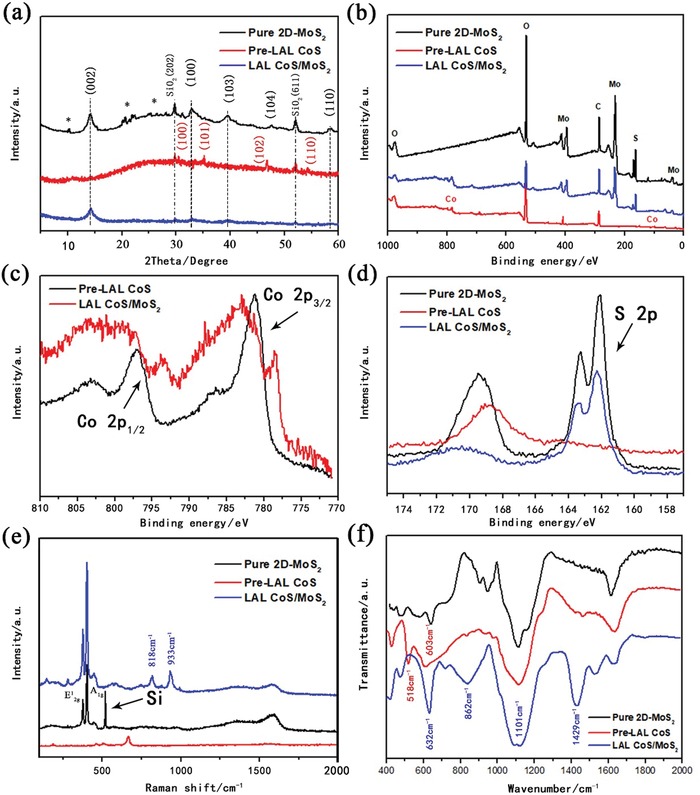
a) XRD patterns of pure 2D‐MoS_2_, pre‐LAL CoS, and CoS/MoS_2_ hybrid. Peaks that marked by * belong to other valences sulfides of molybdenum. b) XPS survey spectrum of 2D‐MoS_2_, pre‐LAL CoS, and CoS/MoS_2_ hybrid. c) XPS spectra of Co 2p. d) XPS spectra of S 2p. e) Raman spectra of pure 2D‐MoS_2_, pre‐LAL CoS, and CoS/MoS_2_ hybrid. f) FTIR spectra of pure 2D‐MoS_2_, pre‐LAL CoS, and CoS/MoS_2_ hybrid.

The crystallographic structures of the obtained samples were examined by X‐ray diffraction (XRD) analysis, as shown in **Figure** **5**a. In the XRD analysis of the pure 2D‐MoS_2_ (black line) and a‐CoS/MoS_2_ hybrid (blue line), one can see that almost all the detected peaks belong to MoS_2_, except the only two peaks located at 29.8° and 51.9°, which can be found in all three patterns, belong to SiO_2_ substrate that be used in the XRD test. Furthermore, there was some extremely low intensity peaks marked with * mainly appear between 20° to 28°, and they were identified as belonging to other valences sulfides of molybdenum (for example, molybdenum trisulfide). Note that, the half width of the peak at 14.5° of a‐CoS/MoS_2_ increase comparing with that of MoS_2_, which matched (002) plane of MoS_2_ structure and imply that the crystal size may decrease in the final sample. Upon this point, we can come to the same conclusion from the TEM testing (Figure 3 and Figure S1, Supporting Information). On the other hand, from the XRD pattern comparison of pre‐LAL CoS and bulk CoS (Figure S2, Supporting Information), we found that the CoS crystallinity significantly decreased after the LAL progress, with the bulk CoS turned to amorphous CoS structure. TEM images and the corresponding SAED pattern analysis shown in Figure 2 also came to the same conclusion. Moreover, after the LAL treatment of CoS/MoS_2_ mixture, the crystallinity of CoS will be further declined, which would make the peaks of CoS hardly be found in the XRD analysis of a‐CoS/MoS_2_ hybrid structure. In fact, in addition to the peak at 14.5° that matched the (002) plane of MoS_2_ (blue line), there is no others peaks can be found in the final products. So, the XRD pattern of a‐CoS/MoS_2_ hybrid (blue line) definitely revealed its unique amorphous/crystal layer heterojunction crystallographic structure among the three productions.

The chemical compositions of a‐CoS/MoS_2_ hybrid were carefully studied by X‐ray photoelectron spectroscopy (XPS). First, the XPS survey spectrum (Figure 5b) confirmed the presence of Co, Mo, and S in the hybrid sheets. Second, it can be seen that the Mo 3d region of 2D‐MoS_2_ and a‐CoS/MoS_2_ (Figure S4, Supporting Information) almost unchanged, which indicated that the chemical environment of Mo has hardly changed and MoS_2_ were not oxidized during the LAL process. Third, The Co 2p region of the CoS/MoS_2_ hybrid (Figure 5c) shows an intense peak at 778 eV and three broad peaks at 782, 793, and 801 eV, respectively, which match the standard signals of Co^2+^ very well, with these signals were attributed to the transitions from the Co 2p_3/2_ and 2p_1/2_ orbitals.^12^, ^19^ Fourth, compared with the analysis of O 1s peaks (Figure 5b), we can confirm that the oxygen appear in the sample mainly exists in the form of organic C—O, which come from the preparation process of the a‐CoS/MoS_2_ hybrid. Therefore, these results further confirmed the Co existing in the a‐CoS/MoS_2_ hybrid is in the form of Co—S. Fifth, the S 2p spectrum of MoS_2_ (Figure 5d) was best fitted for two main peaks of 163 and 169 eV that were existed. However, for the S 2p spectrum of pre‐LAL CoS, the broad band around 169 eV was moved to the lower binding energy and the band at 163 eV was disappeared. According to our speculation, these two signals' change might arise from the breaking of Co—S bond during LAL treatment.^12^ Furthermore, the S 2p region of a‐CoS/MoS_2_ hybrid also exhibits two bands. In addition to the broad and intense band centered around on 163 eV (which was also observed in the pure 2D‐MoS_2_ nanosheets), the band at 169 eV was found move to the higher binding energy state. We supposed that the recovery of the 163 eV signals might arise from sulfur atoms reinteracting with Co ions.

Raman spectra shown in Figure S4 (Supporting Information) and Figure 5e demonstrated the hybrid heterojunction structures further. As indicated in Figure S4 (Supporting Information), all of the peaks of CoS had been changed after LAL progress, which suggested the transformation in structure of the CoS composition. In the Raman spectra of CoS/MoS_2_ hybrid (Figure 5e), except the two peaks at 377 and 403 cm^−1^ that matched ${\text{E}}_{2\text{g}}^{1}$ and A_1g_ peaks of MoS_2_, respectively, there were two unique obvious peaks located at 818 and 933 cm^−1^. As everyone knows, there is no any Raman peaks of pre‐LAL CoS or CoS crystal that can be found at these two locations, and there are no such two peaks that had been mentioned in other kinds of S—Co—Mo bonds in the current literature except our synthesized a‐CoS/MoS_2_ hybrid structure. Therefore, these two signals were likely arise from the synergistic effects of a‐CoS/MoS_2_ hybrid. To investigate the functional groups and bonding, catalysts are subjected to Fourier transform infrared spectroscopy (FTIR) measurement (Figure 5f, Figure S3, Supporting Information). As seen in Figure S3 (Supporting Information), additional peaks are observed after LAL progress. The peaks in the range of 1430–1630 cm^−1^ correspond to bending vibration of hydroxyl group. In both the spectra of pre‐LAL CoS and CoS/MoS_2_ hybrid, the peaks in the range of 603–1429 cm^−1^ corresponded to cobalt sulfide. In the spectra of a‐CoS/MoS_2_ hybrid (Figure 5f), the transformation of peaks at 632 and 1429 cm^−1^ was owing to the synergistic effects of the a‐CoS/MoS_2_ hybrid. Comparing the FTIR spectra of Pre‐LAL CoS and CoS/MoS_2_ hybrid, we can establish a ratiocination: the disappearance of peak at 862 cm^−1^ of Pre‐LAL CoS may be owing to the broken Co—S bond in the LAL process, and the reappearance of this peak in the FTIR spectrum of LAL‐CoS/MoS_2_ means unsaturated S atoms of MoS_2_ may bond with Co atoms again. Note that the XPS analysis of S 2p we mentioned above had also prove this ratiocination. Therefore, the FTIR result became a more definite evidence for the presence of synergistic effects between amorphous CoS nanosheets and multilayered MoS_2_. Beyond that, under these conditions the synergistic effect of a‐CoS/MoS_2_ heterojunction averaging the bond lengths will lead to the transformation of the FTIR peaks.^11^, ^25^ Therefore, all the FTIR results were consistent well with the Raman and XPS analyses.

### 2.1 Catalytic Hydrogen Evolution

**Figure 6:**
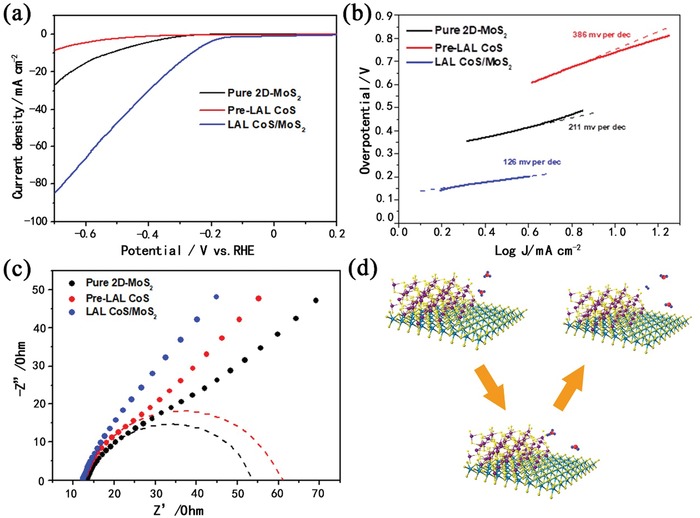
a) Polarization curves for HER of pure 2D‐MoS_2_, pre‐LAL CoS, and CoS/MoS_2_ hybrid. b) Tafel plot for the various catalysts derived from (a). c) EIS Nyquist plot of pure 2D‐MoS_2_, pre‐LAL CoS, and CoS/MoS_2_ hybrid. d) Reaction pathway of HER on CoS/MoS_2_ hybrid according to the Volmer–Heyrovsky route.

To assess the HER electrocatalytic activity, the thin films of various catalysts were prepared on glassy carbon (GC) electrodes in 0.5 m H_2_SO_4_ electrolyte (see the Experimental Section for experimental details). Potentials were measured versus saturated calomel electrode (SCE) and the results were reported versus reversible hydrogen electrode (RHE). A stirrer was kept rotating in acidic electrolyte during the measurements to remove in situ emerged H_2_ bubbles. **Figure** **6**a shows that the a‐CoS/MoS_2_ hybrid has a low η of −147 mV for the HER, corresponding to catalytic H_2_ evolution. By contrast, the pure 2D‐MoS_2_ nanosheets exhibited inferior HER activity with a larger onset potential of 248 mV and lower catalytic current, while pure amorphous CoS nanosheets only affected little HER activity. The HER kinetics of the above catalysts was probed by corresponding Tafel plots (log *j* ∼ η) (Figure 6b). Tafel slope of 126 mV per decade was measured for CoS/MoS_2_ hybrid, which was lower than that of 211 mV per decade for MoS_2_ nanosheets and 386 mV per decade for amorphous CoS, demonstrating the better HER kinetics of a‐CoS/MoS_2_ hybrid. The high electrode kinetic metrics (including onset potential of −147 mV and the Tafel slope of 126 mV per decade) highlight the good H_2_ evolving efficiency of the prepared a‐CoS/MoS_2_ hybrid catalyst. Figure 6c shows Nyquist plots of the pure 2D‐MoS_2_ nanosheets, amorphous CoS, and a‐CoS/MoS_2_ hybrid. *Z*′ is the real impendence and −*Z*″ is the imaginary impedance. The kinetics of electrode reactions for pure 2D‐MoS_2_ nanosheets, amorphous CoS, and a‐CoS/MoS_2_ hybrid were also probed by electrochemical impedance spectroscopy (EIS) technique. The Nyquist plots (*Z*_real_ vs −*Z*_im_) of pure MoS_2_ nanosheets and amorphous CoS both consist of a depressed semicircle in the high‐frequency region (corresponding to charge transfer resistance, *R*_ct_) and a quasisloping line in the low‐frequency region (corresponding to mass transfer resistance), while the Nyquist plot of CoS/MoS_2_ hybrid is nearly a quasisloping line. The almost nonexistented *R*_ct_ value of CoS/MoS_2_ hybrid electrode suggests that it indeed has the highest charge transport efficiency and fastest HER kinetics among the three catalysts.

### 2.2 HER‐Enhanced Mechanism

The experimentally observed high HER activity of the a‐CoS/MoS_2_ hybrid catalyst prompted us to probe the enhanced mechanism. Generally, the value of Δ*G*_H_ is considered as a reliable descriptor of HER activity for many kinds of catalysts.^26^, ^27^, ^28^ As everyone knows, the closer the Δ*G*_H_ of the catalyst is to 0, the better its catalytic activity is. Using density functional theory (DFT) calculations, we can elucidate the thermochemistry of the hydrogen evolution reaction.^26^, ^27^, ^28^ For a chemical process to proceed around room temperature, no reaction step can be carried out with large changes in the free energy. This means the materials that form strong bonds to atomic hydrogen cannot be a good catalysts because the hydrogen release step will be slow.^5^, ^29^ Metals that do not bind to atomic hydrogen are also excluded because here the proton/electron‐transfer step will be thermodynamically uphill and therefore slow. Recently, Hu and co‐workers have found that amorphous material films have higher HER intrinsic activity, that is, the amorphous nature of amorphous films leads to more unsaturated sites.^12^ So this may be the reason why our amorphous CoS films are more active than the single crystals and nanoparticles. Furthermore, Co can promote the HER activity of MoS_2_ by coupling with S‐edges to lower their Δ*G*_H_ to afford a faster proton adsorption kinetics.^11^ For our a‐CoS/MoS_2_ hybrid, the MoS_2_ nanosheets and the coated amorphous CoS heterojunction can bring even more active edge sites, while the CoS chemically interacts with MoS_2_ by forming S—Co bond (Figure 5). Therefore, the amorphous CoS nanosheets, a material with low HER activity by itself, can chemically coupled with MoS_2_ to promote the HER activity. These electrocatalytic synergistic effects together lead to the high HER performance of the a‐CoS/MoS_2_ hybrid catalyst.

For the HER in acidic media, two separate pathways (the Volmer–Tafel or the Volmer–Heyrovsky mechanism) have been proposed for reducing H^+^ to H_2_.^27^, ^28^, ^30^ These two mechanisms involve three principal steps: the Volmer (electrochemical hydrogen adsorption)

(1) $${\text{H}}_{\text{3}}{\text{O}}^{+}+{\text{e}}^{-}\to {\text{H}}_{\text{ads}}+{\text{H}}_{\text{2}}\text{O}$$

the Heyrovsky (electrochemical desorption)

(2) $${\text{H}}_{\text{ads}}+{\text{H}}_{\text{3}}{\text{O}}^{+}+{\text{e}}^{-}\to {\text{H}}_{2}+{\text{H}}_{\text{2}}\text{O}$$

and the Tafel (chemical desorption) reactions

(3) $${\text{H}}_{\text{ads}}+{\text{H}}_{\text{ads}}\to {\text{H}}_{2}$$

The different Tafel slopes point to a unique catalytic property for catalysts. A Tafel slope of 126 mV could suggest that a Heyrovsky‐step‐determined Volmer–Heyrovsky mechanism works in the a‐CoS/MoS_2_ hybrid catalyst (Figure 6d). The semiconducting metallic TMDs span a much wider range of hydrogen adsorption free energies (0.5–2.5 eV) than the range of hydrogen adsorption free energies for metallic surfaces (0.05–1.0 eV).^4^ So we estimate that the hydrogen adsorption free energy of a‐CoS/MoS_2_ is about 2 eV.

## 3 Conclusion

In conclusion, we demonstrate the effective and high‐efficiency LAL progress to synthesize amorphous transition metal chalcogenides metarials and corresponding novel amorphous nanosheets/2D crystal layer hybrid heterojunction catalyst. The synthesized a‐CoS/MoS_2_ catalyst shows higher HER catalytic properties in acidic electrolyte than MoS_2_ nanosheets and amorphous CoS with an onset potential of −147 mV and a much smaller Tafel slope of 126 mV per decade. We believe that our study will facilitate the development of a new way to design and synthesize other novel layered materials with effectively manipulated HER catalytic properties and active functionality structures.

## 4 Experimental Section

*Synthesis of Amorphous CoS Nanosheets and CoS/MoS_2_ Hybrid*: All chemicals were of analytical grade and were used as received without further purification. Amorphous CoS nanosheets were prepared utilizing laser ablating in liquid (LAL) process. Typically, crystalline CoS powders (10.0 mg, Alfa Aesar, 99.99%) were dispersed in a glass bottle (30 mL) containing 10 mL mixed solution with a volume ratio of *V*_IPA_/*V*_DIW_ = 1:1(IPA, isopropanol; DIW, deionized water) by ultrasonic oscillation (5 min). The bottle was then fixed on an ultrasound cleaner with ultrasonic oscillation. A laser beam was irradiated into the suspension without a lens. The laser beam was the second harmonic from a Q‐switched Nd:YAG laser with a wavelength of 532 nm, pulse width of 10 ns, frequency of 10 Hz, and single pulse energy of 550 mJ. The LAL process lasted for 2 h. The product was collected by centrifugation.

To prepare a‐CoS/MoS_2_ hybrid, 5 mg as‐prepared amorphous CoS and 5 mg crystalline 2D‐MoS_2_ nanosheets were dispersed in a glass bottle (30 mL) containing 10 mL mixed solution with a volume ratio of *V*_IPA_/*V*_DIW_ = 1:1 by ultrasonic oscillation (10 min). The bottle was then fixed on a magnetic stirrer with continuous stir. A laser beam was irradiated into the suspension without a lens. The laser beam was the second harmonic from a Q‐switched Nd:YAG laser with a wavelength of 532 nm, pulse width of 10 ns, frequency of 10 Hz, and single pulse energy of 20 mJ. The LAL process lasted for 30 min. The product was collected by centrifugation.

*Characterization*: SEM characterizations were carried out on Quanta 400FEG (FEI Co.Ltd.). TEM, HRTEM, and SAED analyses were carried out on a TF20 Jeol 2100F transmission electron microscope. XRD data were obtained by SmartLab (Rigaku, Japan). Raman spectra were recorded on a Renishaw InVia Plus laser micro‐Raman spectrometer (514.5 nm, 90 mW). FTIR spectra were recorded at Frontier FTIR/NIR/FIR (Frontier, PerkinElmer Inc.). XPS and VB XPS spectra were recorded on an XPS scanning microprobe spectrometer (Escalab 250, Thermo‐VF Scientific).

*Electrocatalytic Study*: The electrochemical test was measured by CHI760E (Chenghua, China) workstation with a three‐electrode electrochemical cell at room temperature. Working electrode was made up of GC (6 mm diameter, 0.2826 cm^2^). The GC electrode was polished to a mirror finish and thoroughly cleaned before use. Graphite rod and SCE were used as counter and reference electrodes, respectively. The potentials reported in this work versus the RHE through RHE calibration is described below.

The preparation method of the working electrodes containing investigated catalysts can be found as follows: 10 mg of catalyst powder was dispersed in 1 mL of 3:1 v/v DIW/isopropanol mixed solvent with 40 µL of Nafion solution (5wt%, Sigma‐Aldrich), then the mixture was ultrasonicated for about 30 min to generate a homogeneous ink. Next, 20 µL of the dispersion was transferred onto the GC disk, leading to the catalyst loading of 0.71 mg cm^−2^. Finally, the as‐prepared catalyst film was dried at room temperature.

## Conflict of Interest

The authors declare no conflict of interest.

## Supporting information

Supporting Information
