# Supplementary material for: Efficient All‐2D Amorphous Cobalt Sulfide Nanosheets/Multilayered Molybdenum Disulfide Hybrid Heterojunction Catalyst for Electrochemical Hydrogen Evolution
Source: Glob Chall. 2019 Dec 9;4(2):1900066. doi: 10.1002/gch2.201900066 (PMC7001116; doi:10.1002/gch2.201900066)
Supplement: Supplementary file 1 — Supporting Information [file GCH2-4-1900066-s001.pdf]

Copyright WILEY-VCH Verlag GmbH & Co. KGaA, 69469 Weinheim, Germany, 2019.

Supporting Information

for *Global Challenges,* DOI: 10.1002/gch2.201900066

Efficient All-2D Amorphous Cobalt Sulfide Nanosheets/ Multilayered Molybdenum Disulfide Hybrid Heterojunction Catalyst for Electrochemical Hydrogen Evolution

Zhenbang Li, Pu Liu, and Guowei Yang**

Supporting Information for

Efficient all-2D amorphous cobalt sulfide nanosheets/multilayered molybdenum disulfide hybrid heterojunction catalyst for electrochemical hydrogen evolution

Z. B. Li, P. Liu *, G. W. Yang **

State Key Laboratory of Optoelectronic Materials and Technologies, Nanotechnology Research Center, School of Materials Science & Engineering, Sun Yat-sen University, Guangzhou 510275, Guangdong, P. R. China

* Corresponding author: liupu5@mail.sysu.edu.cn

** Author to whom correspondence should be addressed: stsygw@mail.sysu.edu.cn

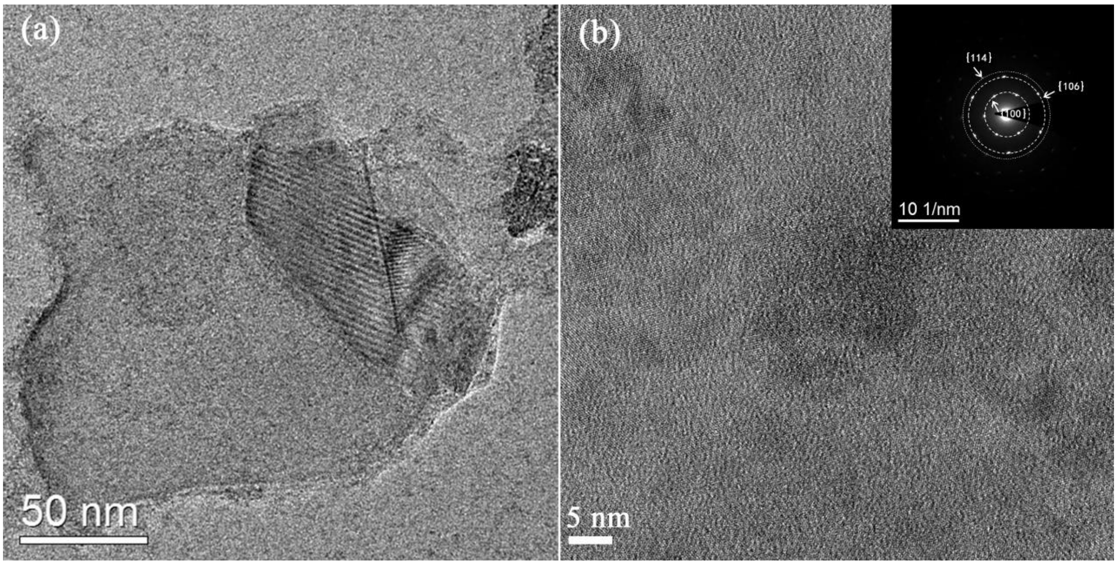

Figure S1. Characterization of 2D-MoS_2. (a) TEM image of 2D-MoS_2. (b) HRTEM image of 2D-MoS_2. The inset shows corresponding SAED pattern.

Figure S1 revealed the compact graphene-like MoS_2 has multilayered structure. Crystal plane{100}, {106}, {114} can be clearly obtained from the SAED pattern

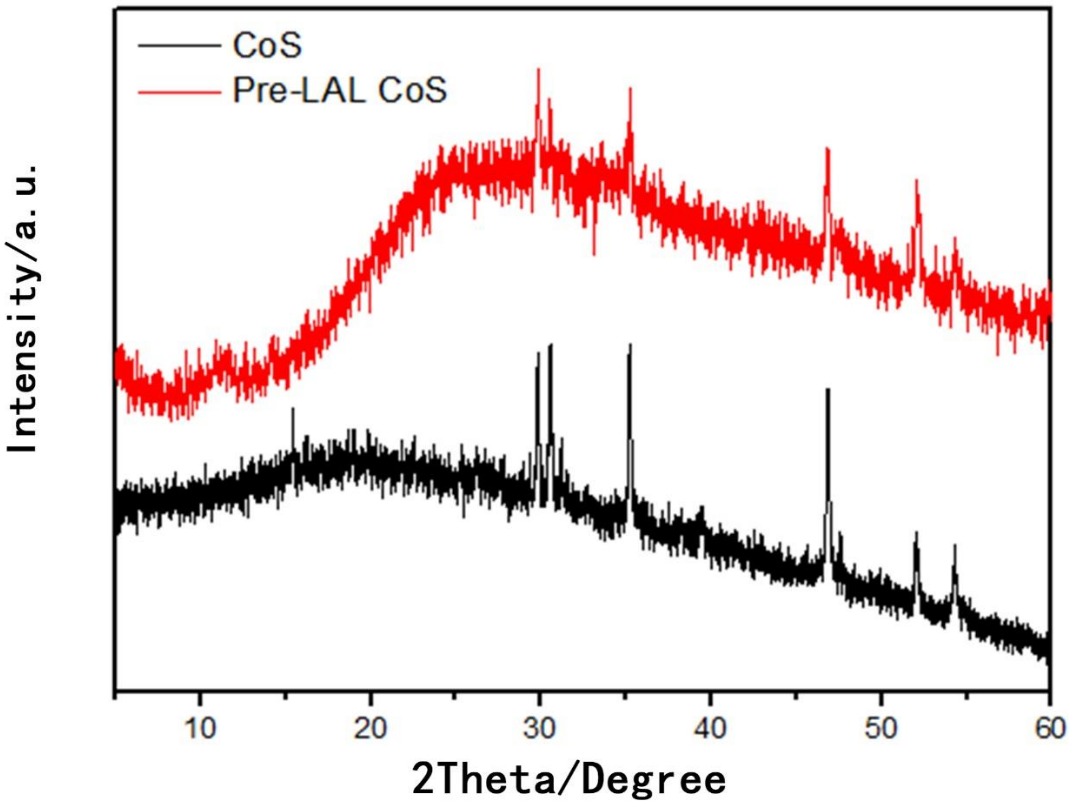

Figure S2. XRD patterns of bulk CoS and Pre-LAL CoS

The XRD showed that some characteristic peaks intensity of Pre-LAL CoS have significant change. XRD pattern and morphology of amorphous CoS clearly suggested its amorphous phase

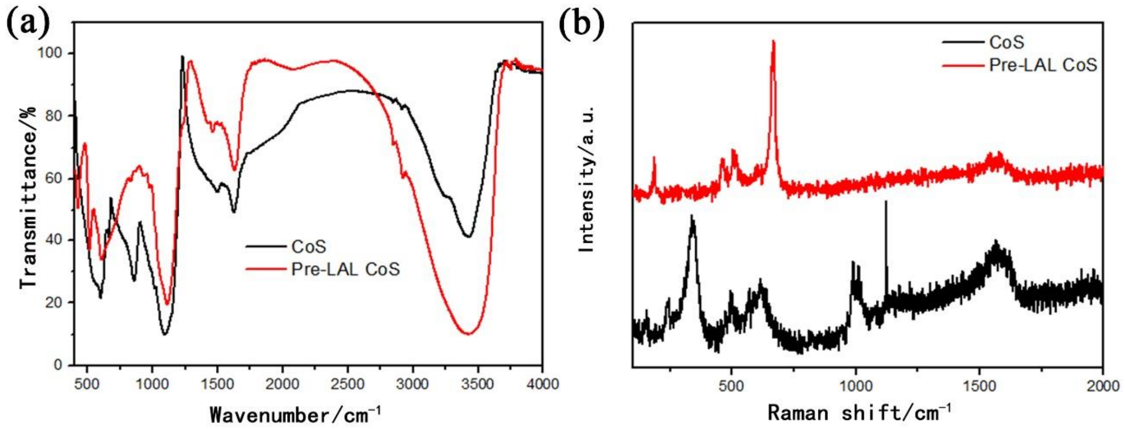

Figure S3. (a) FTIR spectra of bulk CoS and pre-LAL CoS. (b) Raman spectra of bulk CoS, pre-LAL CoS.

The peak at 862cm^{-1} in Figure S3 is disappeared after LAL progress, this may be owing to the broken of Co-S bond in the LAL process.

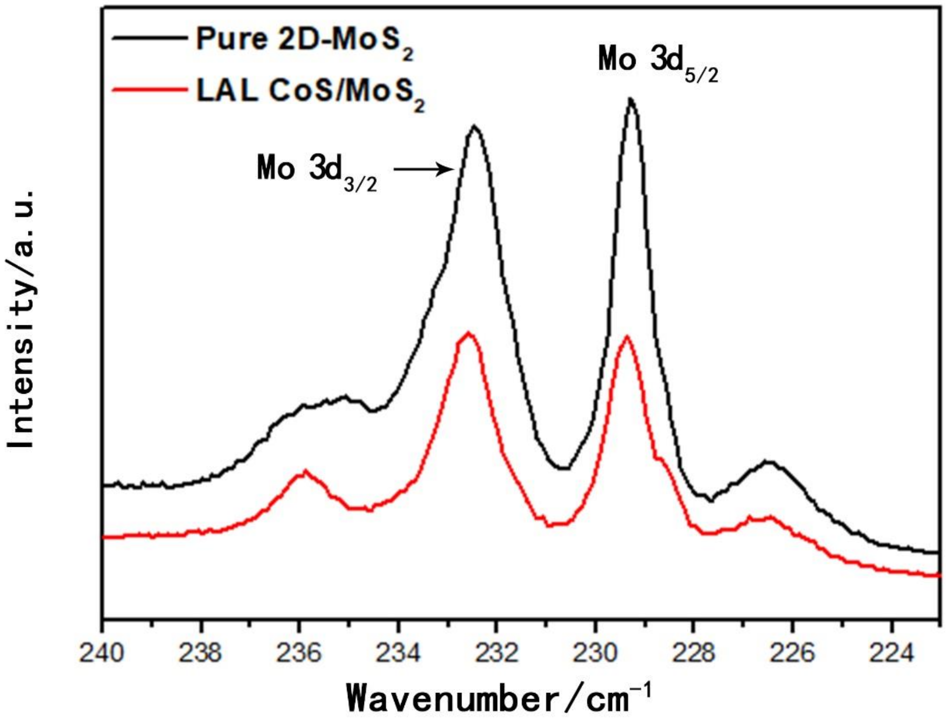

Figure S4. XPS spectra of Mo 3d.

The peaks in XPS spectra of Mo 3d almost unchanged, this indicates that the chemical environment of Mo has hardly changed. Revealed that MoS_2 were not oxidized during LAL process.

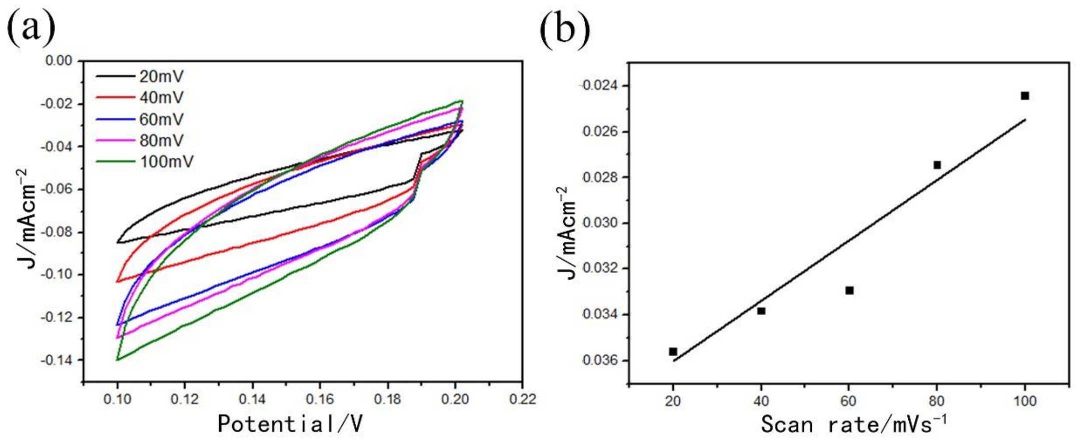

Figure S5. (a) Cyclic voltammograms at E=0.1–0.2 V vs. RHE. (b) scan rate dependence of the current density at E= 0.19 V vs. RHE for 2D-MoS_2.

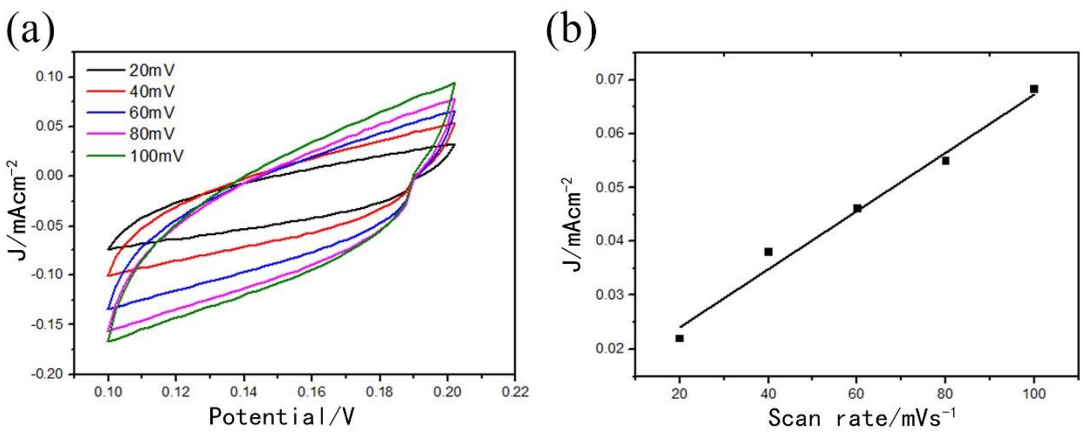

Figure S6. (a) Cyclic voltammograms at E=0.1–0.2 V vs. RHE. (b) scan rate dependence of the current density at E= 0.19 V vs. RHE for Pre-LAL CoS.

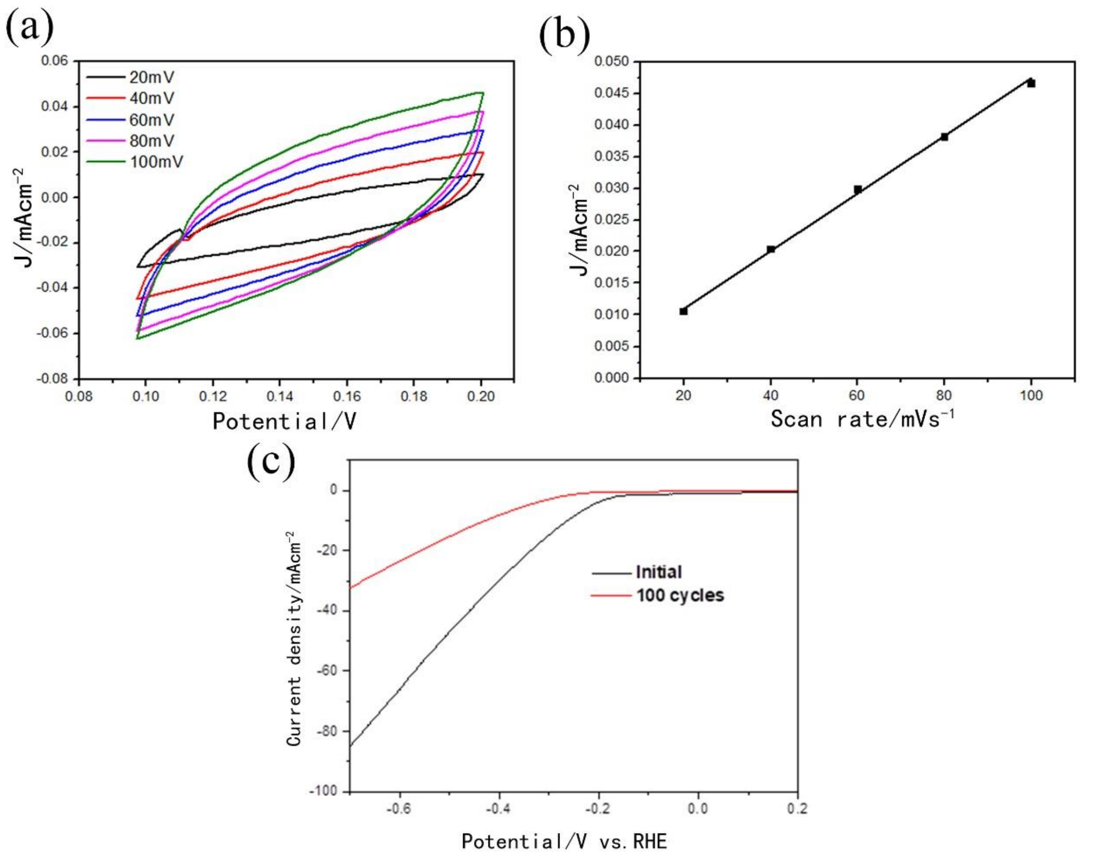

Figure S7. (a) Cyclic voltammograms at E=0.1–0.2 V vs. RHE. (b) scan rate dependence of the current density at E= 0.19 V vs. RHE for CoS/MoS2 hybrid. (c) 100 cycles of LSV test for CoS/MoS2 hybrid.

Figure S5,S6,S7(a), (b) show the C_{dl} of Pre-LAL CoS, 2D-MoS_2 and a-CoS/MoS_2 hybrid, respectively. The slopes of J-Scan rate patterns of Pre-LAL CoS, 2D-MoS_2 and a-CoS/MoS_2 hybrid are 0.0001, 0.0006 and 0.0005, respectively. Compared to the 2D-MoS_2, the a-CoS/MoS_2 hybrid has a little smaller C_{dl}, and note that all of these catalysts are sulfide. All the results show that although CoS/MoS_2 hybrid has almost the same C_{dl} or ECSA with MoS_2, it has better HER catalytic activity. Therefore, the synergistic effects of CoS/MoS2 hybrid must be an important part in HER-enhanced mechanism.

FigureS7(c) shows the stability of CoS/MoS_2. It can be seen from repeated LSV tests that the stability of CoS/MoS_2 is not good as other MoS_2-based catalysts. After 100 cycles, the catalytic activity of the catalyst has decreased significantly. The original crystalline-amorphous heterostructure was not maintained during the HER process, which may resulted in the decrease of HER activity.
